# Esophageal cancers missed at upper endoscopy in Central Norway 2004 to 2021 – A population-based study

**DOI:** 10.1186/s12876-024-03371-z

**Published:** 2024-08-21

**Authors:** Synne Straum, Karoline Wollan, Lars Cato Rekstad, Reidar Fossmark

**Affiliations:** 1 Department of Clinical and Molecular Medicine, Faculty of Medicine, Norwegian University of Science and Technology (NTNU, Trondheim, Norway; 2 Department of Gastrointestinal Surgery, St Olav’s Hospital, Trondheim University Hospital, Trondheim, Norway; 3 Department of Gastroenterology, St Olav’s Hospital, Trondheim University Hospital, Trondheim, Norway

**Keywords:** Esophageal cancer, Quality in endoscopy, Epidemiology, Barrett’s esophagus

## Abstract

**Introduction:**

The incidence of esophageal cancers is increasing in many Western countries and the rate of missed esophageal cancers (MEC) at upper endoscopy is of concern. We aimed to calculate the MEC rate and identify factors associated with MEC.

**Methods:**

This was a retrospective population-based cohort study including 613 patients diagnosed with esophageal cancer in Central Norway 2004–2021. MEC was defined as esophageal cancer diagnosed 6–36 months after a non-diagnostic upper endoscopy. Patient characteristics, tumor localization, histological type and cTNM stage were recorded. Symptoms, endoscopic findings, use of sedation and endoscopists experience at the endoscopy prior to esophageal cancer diagnosis and at the time of diagnosis were recorded. The association between these factors and MEC was assessed.

**Results:**

Forty-nine (8.0%) of 613 cancers were MEC. There was a significant increase in annual numbers of esophageal cancer (*p* < 0.001) as well as of MEC (*p* = 0.009), but MEC rate did not change significantly (*p* = 0.382). The median time from prior upper endoscopy to MEC diagnosis was 22.9 (12.1–28.6) months. MEC patients were older and were diagnosed with disease with a lower cTNM stage and cT category than non-missed cancers, whereas tumor localization and histological type were similar between the groups. The use of sedation or endoscopist experience did not differ between the endoscopy prior to esophageal cancer diagnosis and at the time of diagnosis. High proportions of MEC patients had Barrett’s esophagus (*n* = 25, 51.0%), hiatus hernia (*n* = 26, 53.1%), esophagitis (*n* = 10, 20.4%) or ulceration (*n* = 4, 8.2%). Significant proportions of MECs were diagnosed after inappropriate follow-up of endoscopic Barrett’s esophagus, histological dysplasia or ulcerations.

**Conclusions:**

The annual number of MEC increased during the study period, while the MEC rate remained unchanged. Endoscopic findings related to gastroesophageal reflux disease such as esophagitis and Barrett’s esophagus were identified in a high proportion of patients with subsequent MECs. Cautious follow-up of these patients could potentially reduce MEC-rate.

## Introduction

Esophageal cancer is the eight most common cancer worldwide and esophageal cancer causes the sixth highest number of deaths per year, which makes it one of the most fatal malignancies [1]. Adenocarcinomas and squamous cell carcinomas account for more than 95% of esophageal cancers. In Western populations adenocarcinomas are dominant, with important risk factors such as obesity, smoking and gastroesophageal reflux disease (GERD) [2]. The incidence of esophageal cancer overall in Norway is rising and has increased by 20% over the past two decades [3]. A similar increase in age-standardized incidence rate is also seen in other Western countries over the last three decades, ranging from 6.7 to 86.6% [4]. While the incidence of squamous cell carcinoma has decreased, the incidence of adenocarcinoma is increasing, particularly amongst men [5]. In Norway, adenocarcinomas accounted for 75–80% of the annual cases in 2017 while squamous cell carcinomas (SCC) accounted for approximately 20% [6]. The mortality rate of esophageal cancer is high. The total five-year relative survival of the Norwegian patients including all disease stages was 29.8% for women and 22.2% for men in 2021 [3].

Esophageal cancer is often diagnosed at an advanced stage, the main reason being the lack of early clinical symptoms [7]. Amongst the patients who develop symptoms, the most common presenting symptoms are dysphagia alone, or dysphagia accompanied by unintentional weight loss [2]. Tumor stage at diagnosis is the main prognostic factor, and while the primary tumor may be small, the risk of lymph node metastases increases considerably with submucosal invasion > 1000 μm [8]. An earlier diagnosis is therefore important to improve survival. Unfortunately, small tumors are often asymptomatic and early symptoms may be subtle and unspecific [9]. Esophagogastroduodenoscopy with biopsy is the standard procedure for diagnosing esophageal cancer [10]. Several studies have questioned the efficacy of upper endoscopy for diagnosis of early-stage esophageal neoplasms [9, 11–13]. A population-based study in England found that 7.8% of diagnosed esophageal cancers had undergone negative endoscopy 3 to 36 months prior to diagnosis [12]. Another study showed that missed esophageal cancer (MEC) rate, defined as esophageal cancer detected within 36 months after negative upper endoscopy, was 6.4% [9]. Further studies are important to assess the quality of upper endoscopies in countries with increasing incidence of esophageal cancer as well as to identify risk factors for MEC. The aim of this study was therefore to calculate the rate of missed esophageal cancer as well as to identify factors associated with missed cancers.

## Materials and methods

### Study design, setting, participants and data source

This was a retrospective cohort study of patients with esophageal cancer in Central Norway diagnosed between 01.01.2004 and 31.12.2021. Participants were identified by a search in the Cancer Registry of Norway, using International Classification of Diseases (ICD)-10 codes C15.x. and esophageal cancer cases and histological type reported to the Cancer Registry of Norway were verified by manual review of the electronic medical records. Esophageal cancer was defined as a primary cancer in the esophagus and the initial population-based cohort consisted of 625 patients. Subsequently, clinical and disease variables were extracted from medical records. We performed a search to identify upper endoscopies performed within the time interval 6 to 36 months before the cancer diagnosis using the upper endoscopy Nomesco Classification of Surgical Procedures (NCSP) codes JUD02, JUD05, UJD02 and UJD05. In addition, medical records up to 36 months prior to the esophageal cancer diagnosis were reviewed for previous endoscopy reports. Upper endoscopies in Central Norway from 2004 to 2021 were performed using Olympus endoscopes GIF-160/H180/Q180/HQ190.

### Variables

**Fig. 1:**
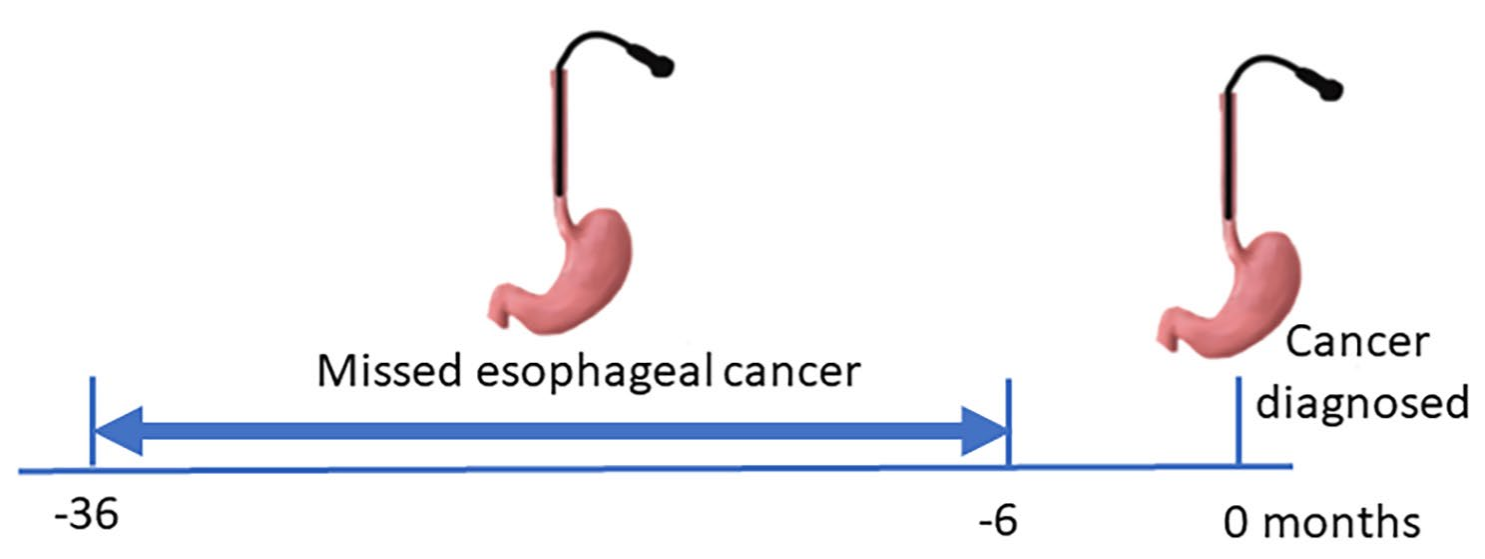
Missed esophageal cancer was defined as cancer diagnosed 6 to 36 months after an upper endoscopy where cancer was not diagnosed

MEC was defined as incident esophageal cancer diagnosed 6 to 36 months after a previous upper endoscopy where cancer was not diagnosed [14] (Fig. 1). Patients who underwent an endoscopy less than 6 months prior to esophageal cancer diagnosis were excluded, as a proportion of such endoscopies were a planned intensified follow-up leading to a diagnosis.

The date of esophageal cancer diagnosis was defined as the date of the upper endoscopy where a lesion was described as suspicious of cancer and biopsied or described as a macroscopic cancer, but insufficiently biopsied and not re-biopsied due to e.g. short life expectancy. In patients where esophageal cancer was found at autopsy, the date of diagnosis equaled the date of death. In patients with histological high-grade dysplasia in biopsies, but the subsequent resection specimen was a histological adenocarcinoma, the date of cancer diagnosis was recorded as the date when high-grade dysplasia was found. The patient group with non-missed cancers was used as control group.

Tumor localization was categorized according to ICD10-codes from C15.3 to 15.9 reported to the Cancer Registry of Norway, giving five different localizations; upper third, middle third, lower third, overlapping lesions and not specified. In patients with unspecified localization (C15.9) the medical records were reviewed to localize the tumor based on endoscopic findings or cross-sectional imaging. Histological types were grouped into four categories: adenocarcinoma, squamous cell carcinoma, neuroendocrine tumor and malignant tumor other than those specified above. Twelve lesions with insufficient evidence of being a primary esophageal malignant tumor or being exceptionally rare (one malignant melanoma and a hemangiosarcoma) were excluded from further analyses and the final study cohort thus consisted of 613 patients. The clinical TNM (cTNM) of each cancer was staged according to the UICC 8th edition [15].

Macroscopic and histological findings at the upper endoscopy preceding the diagnosis of cancer were recorded and classified as normal, tumor, esophagitis, ulceration, Barrett’s esophagus, hiatus hernia and histological low-grade or high-grade dysplasia. The indications for endoscopy were recorded and classified as dysphagia, gastrointestinal bleeding (defined as either occult bleeding with microcytic anemia, overt bleeding or hematemesis), heart burn and unintentional weight loss. The endoscopists experience was classified as either junior endoscopist (under training) or senior endoscopist (specialist). The use of sedation (classified as no sedation, i.v. conscious sedation, deep sedation with propofol or intubation anesthesia) and the hospital level classified as regional (St. Olav’s Hospital, Trondheim University Hospital) or local hospital (all other endoscopy units) were also recorded.

### Statistical methods

Continuous variables were presented as median (inter quartile range (IQR)) or mean ± standard deviation (SD) depending on distribution and groups were compared using the Mann-Whitney or Students t-test. Categorical variables were analyzed by χ² or Fischer exact test to identify differences between groups. Linear regression analysis was used to assess change in annual number of esophageal cancer and MEC during the study period. *P*-values < 0.05 were considered significant. Statistical analyses were conducted using SPSS version 29 (IBM, Armonk, NY, USA).

## Results

Among the 613 patients in the study cohort, 49 (8.0%) patients had undergone an upper endoscopy 6 to 36 months prior to the date of diagnosis, defined as MEC.

### Patient and characteristics

Median age of the entire cohort was 70.5 (63.6–79.3) years, with 461 (75.2%) of the patients being males (Table 1). MEC patients were older than patients with non-missed cancers (74.0 years vs. 70.1 years, *p* = 0.007), whereas sex did not differ significantly between MEC and non-missed cancers.

**Table 1:** Patient demographics and tumor characteristics

	Entire cohort	Missed cancers	Non-missed cancers	*p*-value
Patients, n (%)*	613 (100.0)	49 (8.0)	564 (92.0)	
Age at diagnosis, years				0.007
Median (IQR)	70.5 (63.6–79.1)	74.0 (69.7–81.4)	70.1 (63.4–78.9)	
Male sex, n (%)	461 (75.2)	34 (70.6)	427 (75.1)	0.326
Cancer localization, n (%)				0.225
Upper third	67 (10.9)	7 (14.3)	60 (10.6)	
Middle third	138 (22.5)	12 (24.5)	126 (22.3)	
Lower third	398 (64.9)	28 (57.1)	370 (65.6)	
Overlapping	7 (1.1)	2 (4.1)	5 (0.9)	
Not specified	3 (0.5)	0 (0)	3 (0.5)	
Histology, n (%)				0.372
Adenocarcinoma	354 (57.7)	32 (65.3)	322 (57.1)	
Squamous cell carcinoma	211 (33.4)	16 (32.7)	195 (34.6)	
Neuroendocrine tumor	12 (2.0)	0 (0)	12 (2.1)	
Malignant tumor	36 (5.9)	1 (2.0)	35 (6.2)	
cTNM-stage				0.0003
I	41 (6.7)	10 (20.4)	31 (5.5)	
II	69 (11.2)	9 (18.4)	60 (10.6)	
III	130 (21.2)	11 (22.4)	119 (21.1)	
IV	324 (52.9)	16 (32.7)	308 (54.6)	
IVa	116 (18.9)	6 (12.2)	110 (19.5)	
IVb	208 (33.9)	10 (20.4)	198 (35.1)	
X (unknown)	49 (8.0)	4 (8.2)	45 (8.0)	
cT category				0.0003
T1	43 (7.0)	10 (20.4)	33 (5.9)	
T2	86 (14.0)	10 (20.4)	76 (13.5)	
T3	252 (41.1)	19 (20.2)	233 (41.3)	
T4	131 (21.4)	3 (6.1)	128 (22.7)	
Tx	101 16.5)	7 (14.3)	94 (16.7)	

*Three patients had synchronous cancers which were counted as separate cases. Two patients with synchronous adenocarcinoma and squamous cell carcinoma, one patient with synchronous neuroendocrine carcinoma and adenocarcinoma. IQR: interquartile range; cTNM: clinical TNM, cT: clinical T

### Tumor localization and histological type

The majority (64.9%) of tumors were localized in the lower third of the esophagus. Tumor localization did not differ significantly between MEC and non-missed cancers (*p* = 0.225). Similarly, adenocarcinomas constituted the largest proportion (57.7%) of cancers, but histological types did not differ between MEC and non-missed cancers (*p* = 0.372) (Table 1). The category malignant tumor consisted of macroscopic cancer for various reasons without a certain histological verification (*n* = 12) and unspecified carcinoma (*n* = 18). All patients without a certain histological cancer diagnosis had typical findings at an upper endoscopy and at cross-sectional imaging indicating esophageal cancer – in most cases with advanced disease and / or old age .

### cTNM stage

The majority of patients in the entire cohort had advanced disease with 52.9% being in stage IV, whereof metastatic disease (IVb) was more frequent than stage IV caused by advanced T- and N- category (IVa) (Table 1). A minor proportion of the patients (8.0%) did not have cross-sectional imaging or imaging of insufficient quality for cTNM-staging, however these proportions were equivalent among MEC and non-missed cancers (Table 1). Patients with MEC had a significantly lower disease stage overall at diagnosis compared to non-missed cancers (*p* = 0.0003). This difference was driven by missed cancers being more frequent in stage I (20.4% versus 5.5%) and less frequent in stage IV (32.7% versus 54.6%) at the time of diagnosis, compared to non-missed cancers. The T-category was significantly lower in MEC than in non-missed cancers overall, *p* = 0.0003. This difference was related to MEC being more frequently categorized as T1 (20.4% versus 5.9%) or T2 (20.4% versus 13.5%) and less frequently as T4 (6.1% versus 22.7%) compared to non-missed cancers.

### Symptoms in patients with missed cancers

Alarm symptoms at the upper endoscopy prior to esophageal cancer diagnosis were reported in 24 (49.0%) of the patients. The most common symptom at the prior endoscopy was gastrointestinal (GI)-bleeding, followed by heartburn. There was a significantly higher proportion of patients with alarm symptoms at diagnosis compared to at the prior endoscopy (*p* < 0.001) (Table 2). The most common symptom at diagnosis was dysphagia (51.0%), followed by unintentional weight loss (46.9%). A proportion of patients had a combination of symptoms, the most common combination was dysphagia and unintentional weight loss (*n* = 15, 30.6%).

**Table 2:** Symptoms and findings at upper endoscopy in 49 patients with missed esophageal cancer

	Prior endoscopy	Endoscopy at diagnosis	*p*-value
Alarm symptoms, n (%)			
Any alarm symptom	24 (49.0)	40 (81.6)	< 0.001
Dysphagia	4 (8.2)	25 (51.0)	< 0.001
GI-bleeding	11 (22.4)	5 (10.2)	0.049
Heartburn	6 (12.2)	6 (14.3)	1.000
Weight loss UI*	5 (10.2)	23 (46.9)	< 0.001
Findings, n (%)			
Normal	12 (24.5)		
Tumor		41 (83.7)	
Esophagitis	10 (20.4)	4 (8.2)	
Ulceration	4 (8.2)	4 (8.2)	
Barrett´s esophagus	25 (51.0)		
Hiatus hernia	26 (53.1)		
Low-grade dysplasia	8 (8.2)		
High-grade dysplasia	2 (4.1)		
Sedation, n (%)			1.000
No sedation	37 (75.5)	39 (76.5)	
Conscious sedation	10 (20.4)	9 (17.6)	
Deep sedation	2 (4.1)	1 (2.0)	
Endoscopist, n (%)			1.000
Junior	19 (38.8)	20 (40.8)	
Senior	30 (61.2)	29 (59.2)	

*Weight loss UI = Unintentional weight loss

### Findings at upper endoscopy in patients with missed cancers

The most common findings at the endoscopy prior to the MEC diagnosis was Barrett´s esophagus found in 25 of 49 patients (51.0%) and hiatus hernia found in 26 of 49 patients (53.1%) (Table 2). A total of 40 patient (81.6%) had one or more findings associated with GERD, consisting of hiatus hernia, esophagitis, Barrett’s esophagus, ulceration or various combinations of these findings. Amongst the 25 patients with Barrett’s esophagus, 18 patients also had hiatus hernia. Eight patients (16.3%) had low-grade dysplasia and two patients (4.1%) had high-grade dysplasia in biopsies collected at the prior endoscopy.

Forty-one (83.7%) patients had a macroscopic tumor at the endoscopy at diagnosis. The endoscopic finding in the remaining eight (16.3%) patients was esophagitis or ulceration with or without concomitant Barrett’s esophagus that were histologically verified as cancer. Not all patients with Barrett’s esophagus or ulceration were biopsied at the endoscopy prior to esophageal cancer diagnosis. Twenty-eight (57.1%) patients had either Barrett’s esophagus, esophageal ulcer or both at the prior endoscopy and 21 of these were biopsied. Among the seven patients that were not biopsied six patients had Barrett´s esophagus and one had an esophageal ulcer.

### The association between MEC and endoscopist experience, use of sedation and hospital level

The endoscopies prior to the diagnosis, 19 (38.8%) were performed by junior doctors and 30 (61.2%) by senior doctors. The endoscopy leading to the cancer diagnosis was performed by junior doctors in 20 (40.8%) of the patients, whereas 29 (59.2%) were examined by a senior doctor and these difference were not significant. There was no significant difference between the degree of sedation at the prior endoscopy and at diagnosis (Table 2). Finally, the MEC-rate tended to be higher at the university hospital (10.8%) than at local hospitals (6.5%), but the difference was not significant (*p* = 0.085).

### Time from prior endoscopy to EC diagnosis

The median time from prior endoscopy to diagnoses in the entire MEC-group was 22.9 (12.1–28.6) months. Out of the eight patients with low-grade dysplasia at the prior endoscopy the median time to diagnosis was 20.1 (12.0–33.0) months. Two patients had high-grade dysplasia at the prior endoscopy with median time to EC diagnosis of 12.7 (9.2–16.2) months.

### Increase of MEC per year

**Fig. 2:**
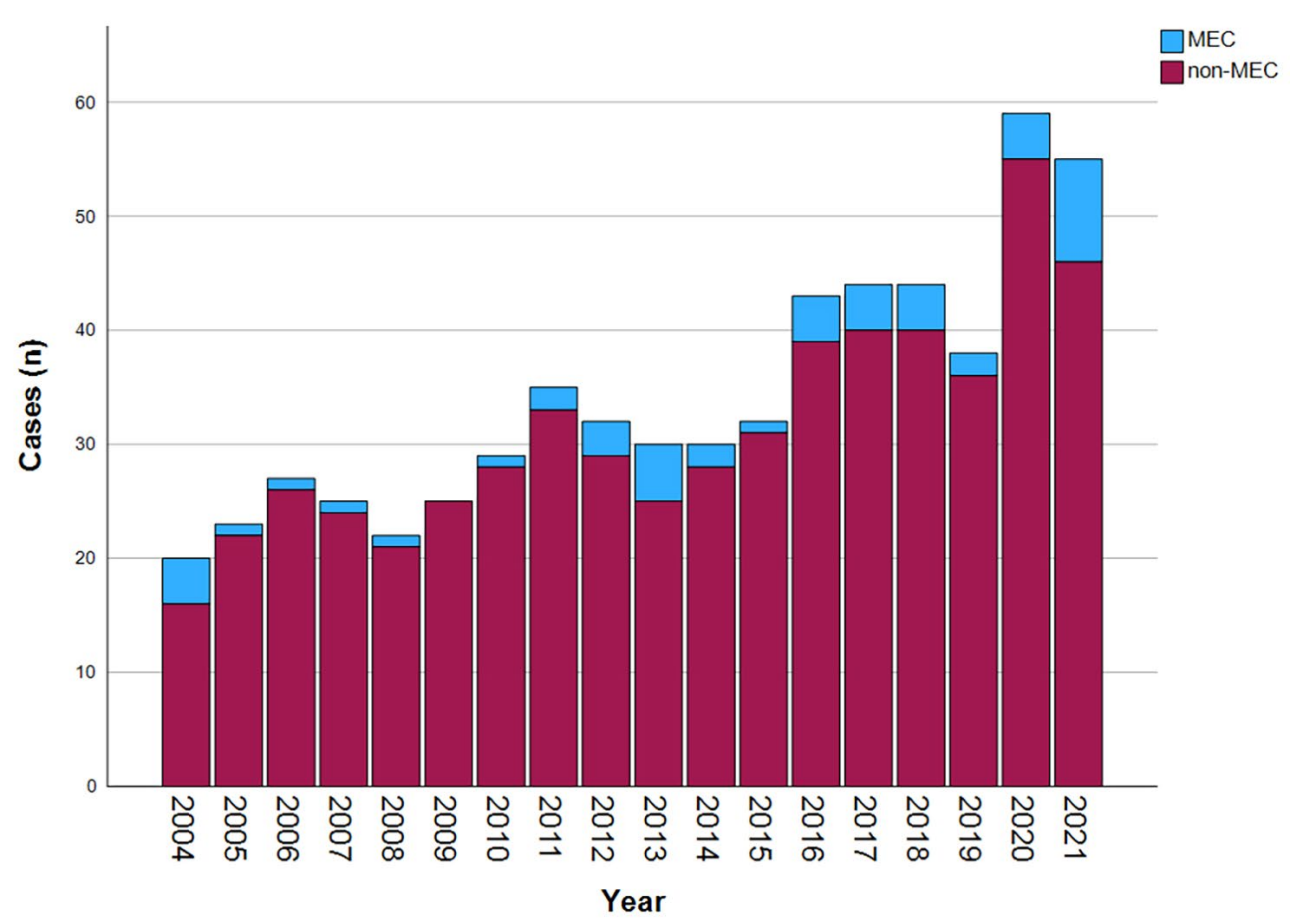
Annual number of missed esophageal cancers (MEC) in comparison to the total number from 2004 to 2021

There was an increase in annual number of esophageal cancer during the study period (*p* < 0.001). There was also an increase in annual number of MEC (*p* = 0.008), whereas the annual MEC rate (MEC/all esophageal cancers) did not change significantly (*p* = 0.382) (Fig. 2).

## Discussion

The rate of MEC in this population-based study in Central Norway was 8.0%. The rate is comparable to other Western studies, with MEC reported to account for 6.4 to 12.7% of all esophageal cancers [9, 11–13]. Patient populations at higher risk of esophageal cancer have also been studied and the incidence of adenocarcinoma identified within one year after a nondiagnostic endoscopy in Barrett’s esophagus patients has been found in the range of 9–25% [11, 16, 17]. Various definitions of MEC have been used in previous studies, in particular has a time from a negative endoscopy to cancer diagnosis from 0 months [9] to 12 months been used as cut-off [18]. In the current study we chose a 6-month cut-off as also done by others [14], as in some patients an endoscopy 3–4 months before the diagnosis of early cancer may have been a planned intensified follow-up of a lesion and represented acceptable clinical practice rather than an entirely missed lesion. For obvious reasons our knowledge about the natural course of untreated cancers is scarce. However, a few studies of patients who have declined treatment, but were still under endoscopic surveillance, have suggested that early esophageal cancers have a relatively slow growth rate compared to larger tumors. The time for a carcinoma in situ of a SCC or adenocarcinoma to progress to an advanced stage appears to be 3–5 years [19, 20] and the majority of early cancers were still considered superficial after 3 years observation time [21, 22]. In our study the selected time limit was 36 months and assuming the progression time as mentioned, most lesions were likely to have been present at the previous endoscopy. The rate of MEC may be used as a marker of quality in upper endoscopies and a standardized definition that balances the risk of under- and overestimating the occurrence of MEC would facilitate comparisons between study populations and is desirable from this point of view. The annual numbers of esophageal cancers and MEC increased considerably during the study period, but the trend towards increasing MEC rate was non-significant. Trends in MEC rate have not been reported in previous publications and are also of interest when monitoring quality in upper endoscopy.

The association between MEC and patient and disease variables was analyzed. The MEC rate did not differ significantly between histological types and this finding correlates to other studies [12]. Although Barrett’s esophagus patients undergo surveillance endoscopies, the proportion of adenocarcinoma in the MEC group was not higher than that of SCC and neuroendocrine tumors, and cancers of either histological type seem to be missed to a similar degree.

We found that patients in the MEC-group were four years older than non-MEC patients at diagnosis. Other studies have found that younger age and female gender was associated with missed cancers [18]. Such differences may reflect the time spent on inspection of the esophagus during an upper endoscopy and may differ between patient populations and be influenced by the indication for endoscopy. It may also reflect that the incidence of esophageal cancer increases with higher age [3]. Cancer localization did not differ significantly between patients with MEC and non-missed cancers. Some studies have suggested that the mid- and upper esophagus are not as well inspected as the lower-third [12, 13]. Chadwick et al. [12] found a greater proportion of patients with upper-third cancer having an endoscopy within one year before diagnosis and highlighted the need for a careful inspection of this area [12, 13].

The cTNM stage of MEC was significantly lower compared to the stage of non-missed cancers. There was a considerable higher proportion of stage I MEC compared to non-missed cancers (21.4% versus 5.5%), but stage IV disease was also far less frequent (32.7% versus 52.9%). These marked differences in disease stage at diagnosis have also been observed also by others [12, 23]. Parallelling the differences in disease stage we also found that MEC were diagnosed in a lower cT-category, with higher frequencies of T1 and T2 tumors and with a lower frequency of T4. These findings may be explained by early-stage cancers being more likely to be overlooked at endoscopy whereas the diagnosis of advanced disease is less to be delayed long enough (3–12 months depending on the definition being used) to be classified as MEC.

At the upper endoscopy prior to MEC diagnosis, a hiatus hernia was described in 27 (52.9%) of our patients. The prevalence of hiatus hernia in a random sample of 1000 adults in Sweden (mean age 53.5 years, 51% female) was 24%, which is lower than in our MEC patients [24]. The proportion in our study is likely to be higher than in a random population, and related to the strong association between hiatus hernia, gastroesophageal reflux and risk of esophageal adenocarcinoma [25]. Among our 27 patients with hiatus hernia, 18 (66.7%) patients had coexisting Barrett’s esophagus. In a large meta-analysis of 33 studies a hiatus hernia was associated with a four-fold increased risk of Barrett’s esophagus of any length, and the increased risk persisted after adjusting for gastroesophageal reflux and body mass index [26]. The sequence from Barrett’s esophagus via increasing degrees of dysplasia to esophageal adenocarcinoma is well established [27] and Barrett’s esophagus patients undergo surveillance with regular upper endoscopies based on risk assessment according to guidelines that evolved during the study period [28]. 51% of MEC patients had Barrett’s esophagus at the endoscopy prior to the esophageal cancer diagnosis and should have been a part of a follow-up program and undergone regular upper endoscopies, which could contribute to a high frequency of Barrett’s esophagus in MEC patients. Approximately one fifth of MECs were diagnosed after delayed and therefore inappropriate follow-up of low-grade or high-grade dysplasia [29] and this seemed to be an important preventable factor with more consistent early follow-up endoscopies within e.g. three months if focal pathology is suspected.

Contrasting surveillance of Barrett’s esophagus [28], there are currently no guidelines regarding follow-up of esophageal ulcers. Although most esophageal ulcers are benign [30], the malignant potential of ulcers in Barrett’s esophagus is well recognized [31]. Four (8.2%) of our patients had an ulcer at the endoscopy at the endoscopy prior to the diagnosis whereof one was not biopsied. Others have also reported that esophageal ulcers were a frequent finding at the upper endoscopy preceding an esophageal cancer diagnosis [12]. In addition to thorough endoscopic inspection, taking four-quadrat biopsies is important for detecting low-grade dysplasia, but also high-grade dysplasia and early invasive carcinoma as part of Barrett´s surveillance endoscopy [32]. There is increasing evidence of slow progression of endoscopically visible dysplasia in patients with Barrett’s esophagus [33]. The importance of this is confirmed by our study, where six (12.2%) of the MEC-patients were diagnosed with cancer after an upper endoscopy where biopsy was taken from a Barrett’s segment without any description of malignancy. Notably, all patients with Barrett’s esophagus or esophageal ulceration at the prior endoscopy were biopsied in 2014–2021, which may indicate higher awareness of the importance of random biopsies in the recent years.

Alarm symptoms at the upper endoscopy prior to the esophageal cancer diagnosis were reported in 24 (49.0%) of MEC patients and were as expected even more common at the time of diagnosis when recorded in 40 (81.6%) (*p* < 0.001). This is consistent with alarm symptoms being associated with more advanced disease. The most frequent symptom at diagnosis was dysphagia (51.0%), followed by unintentional weight loss (46.9%). Tai et al. [34] found that esophageal cancer were missed more frequently when the indication for endoscopy was anemia, and less commonly when performed for dysphagia. In our study GI-bleeding was the most common alarm symptom at the upper endoscopy prior to the esophageal cancer diagnosis. Whether the cause of GI-bleeding at the time of the gastroscopy was related to missed esophageal malignancy is however uncertain.

It is important that the quality of endoscopies corresponds with the prevalence and severity of diseases found in the upper GI tract. The high incidence of colorectal cancer in Western populations has led to a focus on quality of coloscopies aiming to reduce missed colorectal cancers that have been reported to account for 1.8-9.0% of cancers [35–39]. Comparing missed colorectal cancer rates to MEC-rates in Western countries (6.4–12.7%) [9, 11–13], the challenge of MEC during upper endoscopy is possibly even more substantial. Considering the increasing incidence of esophageal cancer, it seems timely to readdress the quality also of esophagogastroscopy [40, 41].

Various factors have been suggested to explain failure to diagnose cancer during upper endoscopy. Such factors include that observed lesions were perceived as benign and therefore not biopsied, collection of an insufficient number of biopsies or lack of targeted biopsies, as well as complete failure to identify a lesion [13]. The sensitivity of upper endoscopy in the diagnosis of early esophageal cancer is likely to have improved during the study period as e.g. enhanced imaging techniques that increase the diagnostic sensitivity have become more available [42, 43]. Interestingly, others have reported that missed upper GI cancers were most often found at the same location as previously described abnormalities, that was either not biopsied or biopsied insufficiently [44]. This could also arise from inappropriate follow-up as discussed previously. The value of a biopsy of findings perceived as benign is emphasized by our study where 16.3% of MEC were proved to be cancer after an upper endoscopy without suspicion of malignancy.

We observed a trend towards higher MEC-rate at the university hospital (10.8%) than at local hospitals (6.5%). A higher number of junior doctors perform endoscopies at university hospitals during training, however, endoscopist experience were not associated with MEC in our cohort and other studies investigating the effect of the endoscopists´ procedural experience on the sensitivity of upper endoscopy have reported conflicted results [9, 34, 44]. Expertise based on continued training seems to be the main determinant of high-quality gastroscopies, rather than professional background or the endoscopists total procedural volume [34].

Use of sedation is one factor that might be expected to improve quality of endoscopy [34]. The common practice in Central Norway during the study period was no use of sedation as only ¼ of the procedures were performed with sedation, being similar to other reports [45]. Only a few previous studies have examined the use of sedation on missed cancer procedures. When comparing sedation rates at the procedure prior to with the procedure at cancer diagnosis there was no difference within MEC patients. This is consistent with a case-control study of esophagogastric cancers in UK, one of few other studies having investigated the use of sedation in MEC procedures [34].

Strengths of the study included that it was a regional cohort study and included all esophageal cancer diagnosed in Central Norway during the study period. Furthermore, the follow-up was complete. However, the study had a retrospective design with inherent limitations for instance concerning findings at endoscopies and symptoms described in endoscopy reports. The classification of malignant tumors in the gastroesophageal junction changed during the study period and Siewert type II cardia cancers were included into the ICD-10 code C15.5 from 2017. This may have contributed to the observed increase in annual esophageal cancer numbers, whereas the MEC-rate did not change during the study period. The follow-up of Barrett´s esophagus has been heterogeneous during the study period parallelling the differences in multiple international guidelines and the reported MEC rate cannot be related to one specific follow-up regimen. Finally, the reported rate of MEC is a minimum rate as additional endoscopies performed elsewhere may not have been identified.

## Conclusions

Esophageal cancer was missed at a prior endoscopy in at least 8.0% of the patients in this population-based study, which is similar to other Western studies. There was no association between MEC and histological type or localization and endoscopic examination of the entire esophagus is necessary to avoid missing subtle lesions. The use of sedation or endoscopist experience did not appear to influence on MEC. A significant proportion of MECs were diagnosed after inappropriate follow-up of endoscopic Barrett’s esophagus, histological dysplasia or ulceration and these were identified as potentially preventable causes of MEC. Awareness of MEC and associated risk factors, more consistent biopsy of ulcerations, focal lesions and Barrett’s esophagus and earlier follow-up endoscopies in patients with such changes could reduce the MEC rate.

## Data Availability

The data that support the findings of this study are available from the Norwegian Cancer Registry, but restrictions apply to the availability of these data, which were used under license for the current study, and so are not publicly available. Data are however available from the authors upon reasonable request and with permission of Regional Committee for Medical and Health Research Ethics of Central Norway. Questions concerning data may be directed to the corresponding author, Reidar Fossmark, email reidar.fossmark@ntnu.no.
